# A targeted mutational strategy aiding in generating antisense RNA to knockdown the *Ehrlichia chaffeensis* P28-outer membrane protein 19 expression

**DOI:** 10.1128/jb.00334-26

**Published:** 2026-08-04

**Authors:** Xishuai Tong, Dominica Ferm, Huitao Liu, Ying Wang, Chandramouli Kondethimmanahalli, Roman R. Ganta

**Affiliations:** 1 Center of Excellence for Vector-Borne Diseases, Department of Diagnostic Medicine/Pathobiology, College of Veterinary Medicine, Kansas State University 70725, Manhattan, Kansas, USA; 2 Department of Pathobiology and Integrative Biomedical Sciences, Bond Life Sciences Center, College of Veterinary Medicine, University of Missouri 70735, Columbia, Missouri, USA; University of Notre Dame, Notre Dame, Indiana, USA

**Keywords:** molecular genetics, *Ehrlichia chaffeensis*, Rickettsiales, tick-borne pathogen, *Anaplasmataceae*, targeted mutagenesis, antisense RNA

## Abstract

Obligate intracellular pathogenic bacteria belonging to the order Rickettsiales include several important emerging pathogens causing major health and economic impact to humans, companion animals, and agricultural animals. Despite some recent progress, the lack of well-established genetic manipulation methods for diverse research applications remains a challenge. We recently reported the establishment of targeted mutagenesis to disrupt genes in *Ehrlichia* and *Anaplasma* species. Many essential genes of the obligate pathogens, such as *Ehrlichia chaffeensis*, are likely refractory to targeted mutagenesis; thus, we developed a novel targeted mutational approach leading to expression of an antisense RNA facilitating the knockdown of p28-Omp19 protein expression from *ECH_1143*. This gene was selected as its encoded protein is among the highly immunogenic proteins of *E. chaffeensis*, and our prior efforts to generate a loss-of-function mutation were unsuccessful. This method involved introducing a mutation at a distal genomic location within the *E. chaffeensis* genome, allowing generating a 209-nucleotide-long antisense RNA that is complementary to *ECH_1143* sense mRNA expressed from the same gene promoter, duplicated in the genome during mutagenesis. The mutational strategy was designed to retain the surrounding genomic regions unaltered. The antisense knockdown mutation resulted in a reduction of p28-Omp19 expression relative to wild-type *E. chaffeensis* during its replication in a macrophage cell line, where the gene expression is known to occur, and the mutation caused a fitness defect. We anticipate that this novel mutational strategy will be broadly applicable to facilitate investigating essential genes of obligate intracellular bacterial pathogens.

**IMPORTANCE:**

We developed an innovative targeted mutagenesis approach in the obligate intracellular bacterium *Ehrlichia chaffeensis* to express specific antisense RNA (asRNA) to knockdown protein synthesis from a gene. The mutational strategy was carefully designed to retain the genomic sequences surrounding the mutational site unaltered. Our data demonstrate that this asRNA system effectively reduces protein synthesis by the unique mutational strategy and is ideal for investigating genes critical for the bacterial replication *in vitro*. This innovative molecular tool facilitates research in investigating essential gene research in *E. chaffeensis* and holds strong potential to extend similar research on other bacterial pathogens having reduced genomes.

## INTRODUCTION

Obligate intracellular pathogenic bacteria are responsible for causing diseases in millions of humans and several vertebrates worldwide (1, 2). A well-developed targeted mutagenesis system supporting research on obligate intracellular bacteria belonging to the order Rickettsiales within the genera *Ehrlichia*, *Anaplasma*, *Neorickettsia*, *Rickettsia*, and *Orientia* continue to be a major limiting factor for research progress. Several pathogens that belong to these genera are responsible for causing emerging and reemerging tick-borne diseases in humans and various vertebrate animals (1, 3, 4). The pathogens have extensive genome reductions (5–7) and retain predominantly genes critical primarily for intracellular growth adaptation, which creates a unique challenge for defining molecular genetics broadly applicable for diverse research applications. Furthermore, *Anaplasmataceae* family bacteria naturally lack plasmids, thus limiting the ability to use a plasmid in manipulating bacterial gene expression for pathogens of the genera *Anaplasma*, *Ehrlichia*, and *Neorickettsia*. Despite recent progress in using Himar1 random mutagenesis and the development of targeted mutations in Rickettsiales, the probability of generating targeted mutations that cause complete loss of a gene function remains very low (8–12). To date, only a limited number of targeted mutations are described in defining the functional characterization in rickettsial pathogens, including the genera *Anaplasma*, *Ehrlichia*, and *Rickettsia* (8–10, 12–14). Thus, it is evident that additional methodological advances are necessary in developing molecular tools for promoting research on rickettsial pathogens.

Recently, we reported the development of targeted mutagenesis methods that work well for two *Anaplasmataceae* family pathogens: *Anaplasma marginale* and *Ehrlichia chaffeensis* (8–11). We also demonstrated functional disruption and restoration mutations in *E. chaffeensis* (11). We reasoned that, due to the pathogen's reduced genome(s), the majority of the genes are conceivably essential for the bacterial intracellular adaptation, and thus intractable to mutations resulting in a complete loss of functional disruption or ablation. Therefore, we opted to develop a targeted mutagenesis strategy to interfere with the translation of messenger RNA (mRNA) by simultaneously expressing a portion of the coding sequence as an antisense RNA (asRNA), leading to the complementary binding with sense mRNA and reducing protein expression.

Typically, asRNA molecules interact with sense mRNA through complementary base pairing to block translation and downregulate gene expression (15). This mechanism of gene regulation is documented across diverse organisms, including those belonging to archaea and eukaryotes (16–19). Exogenous synthetic antisense oligonucleotide (ASO) production can serve as a powerful tool for studying transient protein knockdown at the post-transcriptional level by hybridizing with the sense mRNA, leading to either mRNA degradation through a ribonuclease-mediated cleavage or steric hindrance of translation by blocking the interaction of mRNA with ribosomes (20, 21). The use of exogenous synthetic ASO is not ideal, as it is typically short-lived and is highly susceptible to nuclease degradation, resulting in a possible limited transient suppression of the target mRNA translation (20). Furthermore, application of exogenous synthetic ASO transfection can be an added challenge for investigations focused on obligate intracellular bacterial organisms, such as for Rickettsiales, due to their intracellular residence within a host cell phagosome or within the cytoplasmic space. A plasmid-based expression of constitutive asRNA production has the potential to provide a continuous supply of asRNA, allowing rapid downregulation of targeted gene expression by inhibiting translation (22, 23). Although our previous work has shown that uptake of genetic material by these organisms is possible (10, 11, 24), the use of a plasmid-based knockdown is not possible for use in Anaplasmataceae family pathogens, such as *E. chaffeensis*, because they naturally lack endogenous plasmids (25).

In the current study, we developed an innovative targeted mutagenesis approach in *E. chaffeensis* to express a 209-nucleotide-long asRNA, which is designed to bind the protein-coding sequence of the sense RNA and knockdown protein synthesis from *ECH_1143*. We selected this gene as its encoded protein, p28-Omp19, is one of the most abundantly expressed immunogenic proteins of *E. chaffeensis* (26), and moreover, our efforts in generating a loss-of-function mutation were unsuccessful. We created an insertion mutation at a remote genomic location to produce the *trans*-acting asRNA that is also made from the duplicated same gene (*ECH_1143*) promoter to reduce p28-Omp19 protein expression. Furthermore, the mutational strategy was carefully designed to restore the genomic sequences surrounding the mutational site as unaltered. The data presented in this manuscript demonstrate that the asRNA generation by the unique mutational strategy is ideal for investigating genes critical for bacterial replication *in vitro*.

## RESULTS

### Construction of a recombinant plasmid aided in generating an asRNA mutation in the *E. chaffeensis* genome

**Fig 1:**
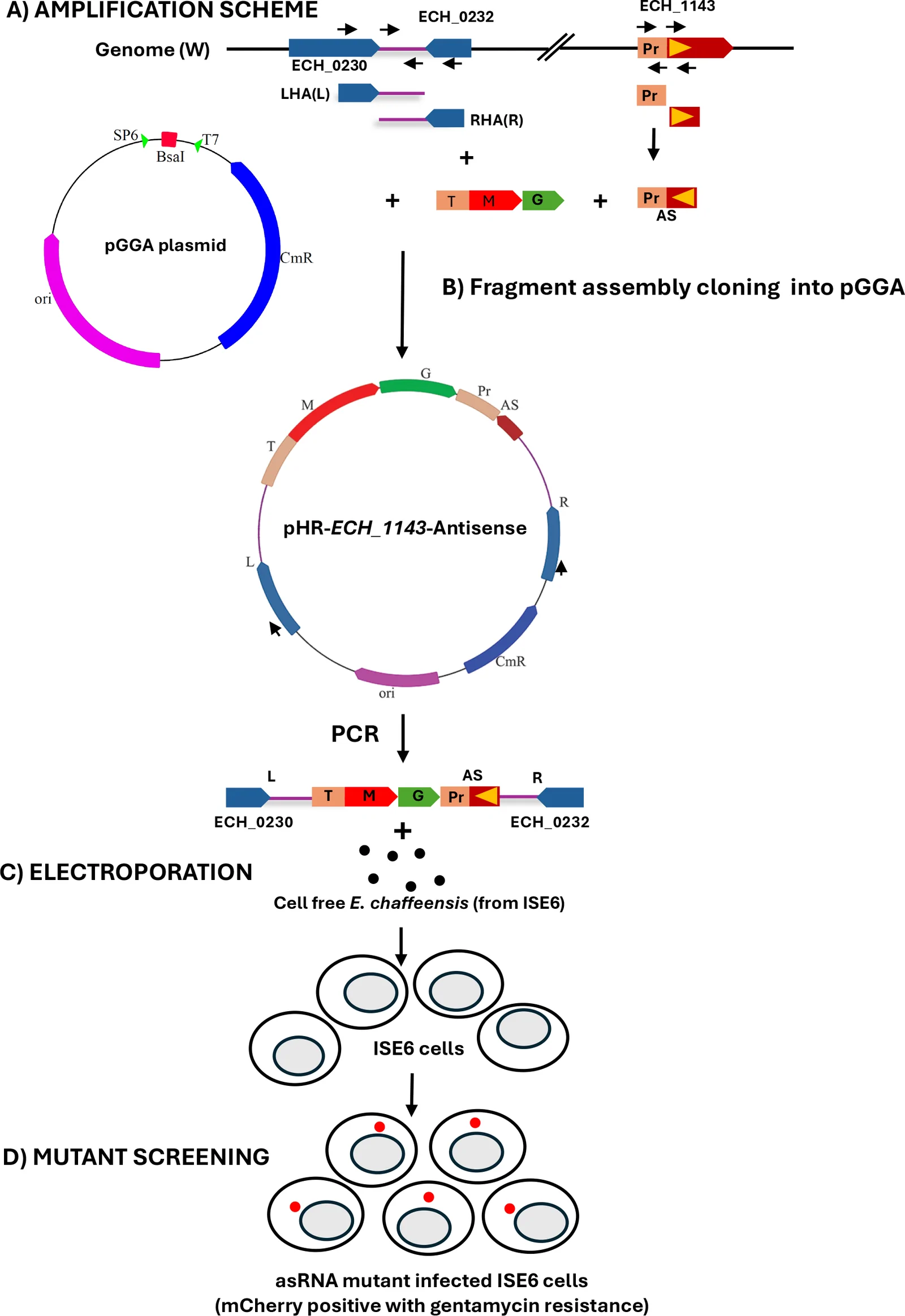
Schematic overview of the *E. chaffeensis ECH_1143* antisense construct generation and its use in creating the asRNA mutation. (**A**) The amplification scheme for the left and homology arms (L and R, respectively) included in generating the same segment of 3′ noncoding region of *ECH_0230* to be positioned at the 3′ and 5′ of the amplified segments, respectively. The *ECH_1143* promoter segment and the first 209 base pairs of the open reading frame (ORF) were amplified separately and combined with selecting the ligated ORF segment in reverse orientation relative to the promoter segment. Tuf (T), mCherry ORF (M), and gentamicin resistance cassette (G) were obtained from a previously prepared recombinant construct (10). (**B**) Fragment assembly cloning of all the fragments into pGGA plasmid in the chosen order was accomplished using a Golden Gateway assembly cloning kit to generate the final recombinant plasmid; pHR-*ECH_1143*-Antisense. (**C**) Electroporation. The mutagenesis segment spanning from L to R was generated by PCR and used to transform ISE6 tick cell culture-derived *E. chaffeensis* organisms. (**D**) Mutant screening to select the mutant in the tick cells was accomplished by screening for the cultures expressing mCherry and resisting gentamicin.

**Fig 2:**
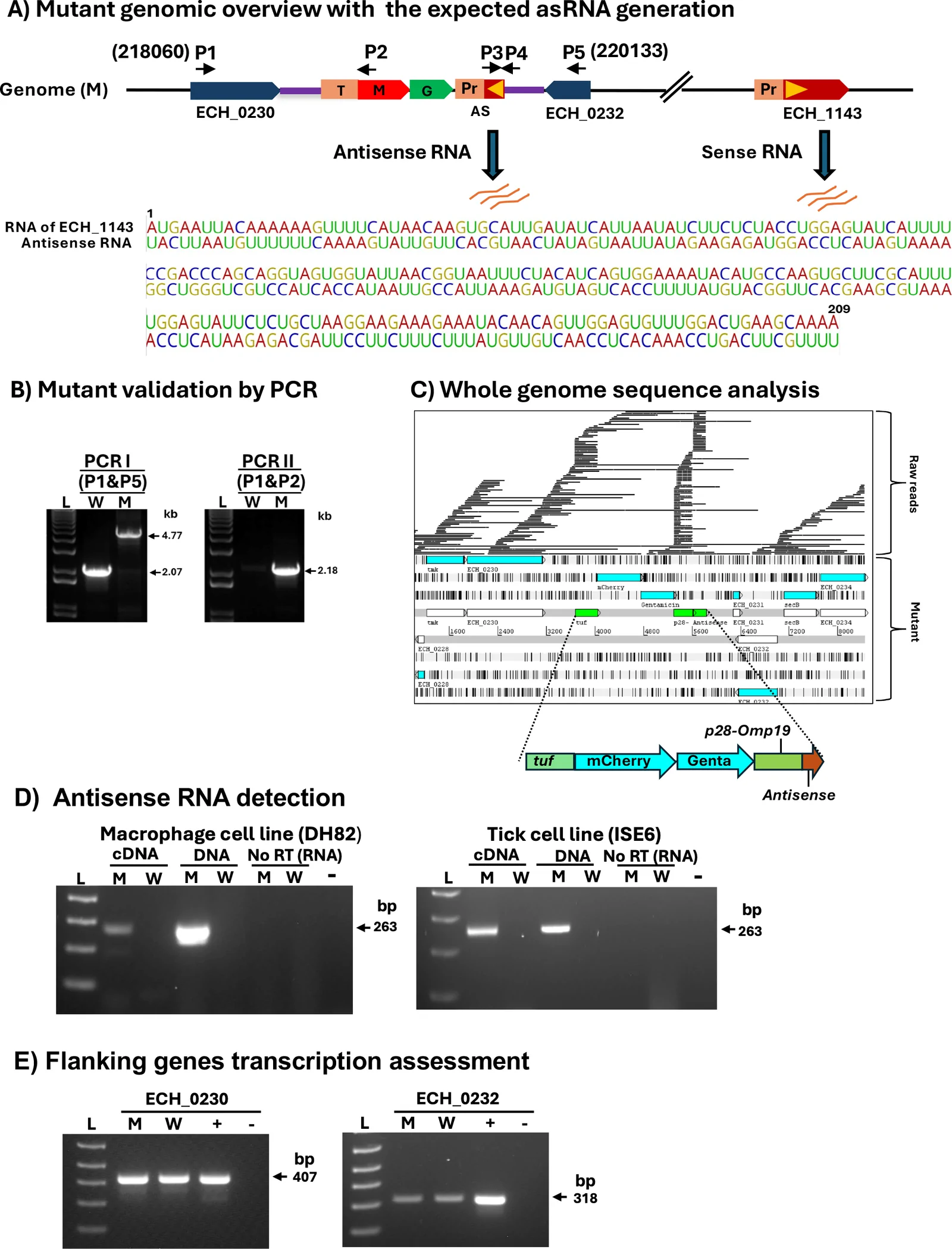
Confirmation of the mutant *E. chaffeensis* by PCR and whole-genome sequencing. (**A**) Schematic view of the duplex RNA strand formation of asRNA with mRNA of *ECH_1143*. The entire predicted asRNA and sense RNA forming hybrids are presented in the image. (**B**) PCR analysis confirming the mutation generation. Primers annealing upstream (P1) and downstream (P5) of the mutation insertion region (genomic coordinates identified) were used to amplify DNA segments by PCR I, which were then resolved on an agarose gel; the expected larger 4.77 kb product was evident from the mutant genomic DNA (M) compared to the smaller 2.07 kb from wild-type genomic DNA (W). (L, 1 kb plus DNA ladder). Similarly, PCR II using primers P1 and P2 targeted to the insertion region and genomic region upstream of the insertion region yielded the expected amplicon of 2.18 kb only from the mutant. (**C**) Nanopore whole-genome sequencing reads were used to assemble the genome of the antisense mutant. The insertion segment was identified at the anticipated region within the *E. chaffeensis* genome. (**D**) Transcriptional analysis confirming the *ECH_1143* asRNA. The presence of asRNA was assessed by RT-PCR using RNA isolated from wild-type (W) and mutant (M) *E. chaffeensis* in the macrophage cell line and tick cell line. RNA samples were reverse transcribed to generate cDNA using primer P4, which was designed to anneal downstream of the asRNA sequence, which was expected to be present only in the asRNA mutant. The cDNA was used as the template for PCR amplification with primers P3 and P4 yielding the expected amplicon of 263 bp. Genomic DNA and RNA with no reverse transcription step from both wild-type and mutant *E. chaffeensis* were included as controls, along with a no-template reaction (−). DNA marker (1 kb Plus DNA ladder) (L) was used to identify specific size amplicon. The asRNA amplicon was detected in the cDNA from the mutant, but not in the cDNA from wild type. Mutant genomic DNA served as the positive control and similarly genomic DNA from wild-type *E. chaffeensis* served as a negative control along with a no-template negative control. RNA-only controls (with no reverse transcription steps) also yielded the anticipated negative results. (**E**) The impact of *ECH_1143* antisense construct insertion on the RNA expression of upstream (*ECH_0230*) and downstream (*ECH_0232*) genes. Transcriptional analysis of RNA isolated from asRNA mutant (M) and wild-type (W) *E. chaffeensis* in macrophage cells was conducted by RT-PCR targeting *ECH_0230* and *ECH_0232*. RNA input was normalized based on 16S rRNA levels quantified by qRT-PCR. Transcript levels of the target genes were then assessed by semi-quantitative RT-PCR (30 cycles). Wild-type genomic DNA served as a positive control (+), and a no-template reaction was included as a negative control (−). DNA marker (1 kb Plus DNA ladder) (L) was used to verify the expected amplicon sizes. Expression of *ECH_0230* and *ECH_0232* was similar for wild-type and mutant *E. chaffeensis*.

We selected *E. chaffeensis ECH_1143* for generating an asRNA mutation for its protein expression knockdown because it is one of the 22 gene paralogs encoding the abundantly expressed and highly immunogenic protein, p28-Omp19 (27–30), and our efforts to generate targeted loss-of-function mutation were unsuccessful. A recombinant plasmid with pGGA plasmid backbone was created by following standard molecular cloning methods to insert multiple segments in the following order (Fig. 1): (i) The left homology arm (L) spanning part of *ECH_0230* coding region and the entire noncoding region located downstream of *ECH_0230*; (ii) *E. chaffeensis tuf* gene promoter (8) (T); (iii) mCherry gene coding sequence (11) (M); (iv) *E. chaffeensis* codon-optimized gentamicin resistance gene coding sequence (11) (G); (v) the entire noncoding DNA segment located downstream of *ECH_1142* and upstream of *ECH_1143*, plus the first 209 base pair segment of the *ECH_1143* protein coding sequence starting from the initiation codon, cloned in the reverse orientation (AS); and (vi) the right homology arm (R) spanning the entire downstream noncoding region of *ECH_0230* and a 3′ end segment of the *ECH_0232* coding region. The duplication of the entire noncoding region downstream of *ECH_0230* was necessary to restore the *E. chaffeensis* genomic sequences as unaltered during mutation development. We selected the mutation insertion site based on our previous demonstration of generating a mutation near this location (8). Linear fragments spanning from L to R, along with the rest of the inserted segments generated by PCR and 3 μg of the purified amplicon, were then used to electroporate tick cell culture (ISE6)-derived *E. chaffeensis* organisms and subsequently reinfected the tick cells (11). Cultures expressing mCherry and resisting gentamicin in the culture media were observed after about 3 weeks of *in vitro* growth at 34°C. A schematic view of the 209-nucleotide-long asRNA generation and its anticipated interaction with the sense mRNA of *ECH_1143* is depicted in Fig. 2A. Two different PCRs were performed to confirm the mutational generation, besides resisting gentamicin in the culture media and testing positive for mCherry expression: PCR I using primers annealing upstream and downstream of the insertion site (primers P1 and P5), which yielded the expected amplicon of 4.77 kb with the mutant genomic DNA as the template, whereas wild-type *E. chaffeensis* genomic DNA yielded the smaller estimated 2.07 kb amplicon (Fig. 2B, left panel). Similarly, a second PCR (PCR II) targeting the insertion region and genomic region upstream of the insertion site (primers P1 and P2) generated the expected amplicon (2.18 kb) only with the mutant genomic DNA as the template, but not with wild-type genomic DNA (Fig. 2B, right panel). Further, whole-genome sequence analysis was performed independently to confirm the presence of the inserted sequences at the desired target site (Fig. 2C). We designed an RT-PCR assay to determine if the novel mutation indeed resulted in generating the anticipated asRNA (Fig. 2D). The assessment was done using RNA recovered from the mutant and wild-type *E. chaffeensis* cultured in macrophage and tick cell lines. Independent of the cell lines used, cDNA specific to the asRNA was detected only in the mutant RNA but not in the wild-type *E. chaffeensis* RNA. To define whether the asRNA mutation insertion 3′ to *ECH_0230* had any impact on the neighboring genes, we performed semi-quantitative reverse transcription-PCR (RT-PCR) analysis (31, 32) targeting the transcripts of *ECH_0230* and *ECH_0232*. The transcript levels for both genes were very similar in the mutant and wild-type *E. chaffeensis* (Fig. 2E).

### Antisense mutation resulted in a reduction of p28-Omp19 protein expression in *E. chaffeensis* cultured in the macrophage cell line

We assessed the p28-Omp19 expression by two independent methods: confocal microscopy and Western blot analysis.

#### Confocal microscopy assessment revealed decreased p28-Omp19 protein expression in macrophage cultures

**Fig 3:**
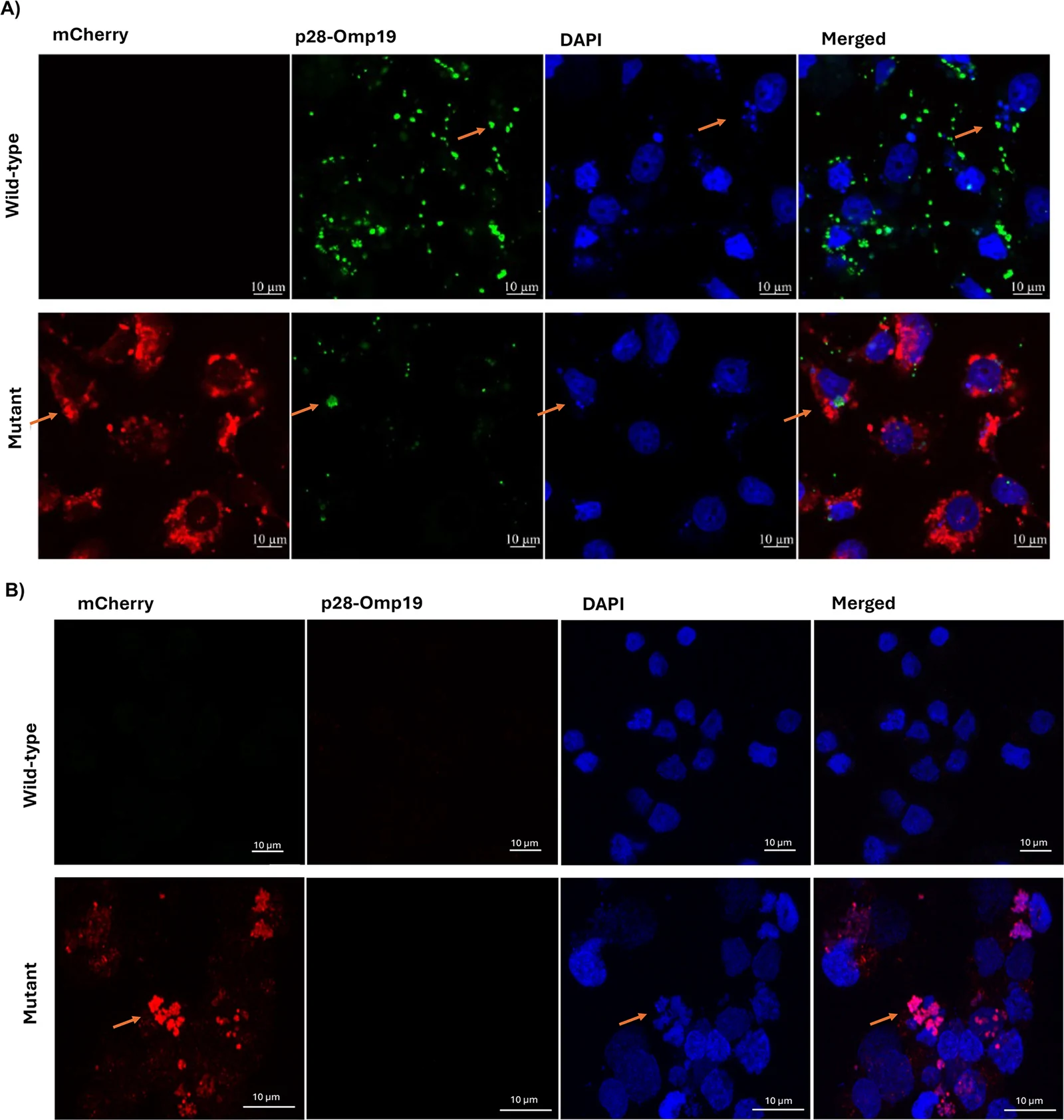
The impact of the *E. chaffeensis ECH_1143* antisense mutation on p28-Omp19 protein expression evaluated in different host cells by confocal microscopy. (**A**) The mutation resulted in a significant reduction of p28-Omp19 expression in the macrophage cell line compared with the wild-type infection. (**B**) P28-Omp19 was not evident in either wild type or mutant when cultured in the tick cell line. Canine macrophage cell line (DH82) and tick cell line (ISE6) were infected with host cell-free wild-type or mutant *E. chaffeensis* and monitored when infection reached about 80% of cells infected. The infected cells were then immunostained using the monoclonal antibody specific to p28-Omp19 (green), while the host and bacterial nuclei were stained with DAPI (blue) (identified with arrows). Mutant bacteria expressing the mCherry (red) fluorescent protein under the constitutively active *tuf* promoter aided in monitoring total bacterial infection.

The p28-Omp19 expression was assessed by confocal microscopy using a p28-Omp19-specific monoclonal antibody, mAb 18.1 (33), for the asRNA mutant in the macrophage and tick cell cultures harvested when infectivity of the cultures was about 80% and compared with wild-type *E. chaffeensis* organisms cultured similarly (Fig. 3). The mutant *E. chaffeensis* exhibited constitutive expression of mCherry (the mutant-specific expressed marker protein) and a considerable reduction in p28-Omp19 expression compared to the wild type in the canine macrophage line (DH82) (Fig. 3A). Consistent with prior published reports, which demonstrate the lack of this protein expression (29, 30), we found no evidence of p28-Omp19 in either wild-type or in the asRNA mutant when cultured in ISE6 tick cells (Fig. 3B).

#### Western blot analysis confirmed the reduction in p28-Omp19 expression in macrophage cultures

**Fig 4:**
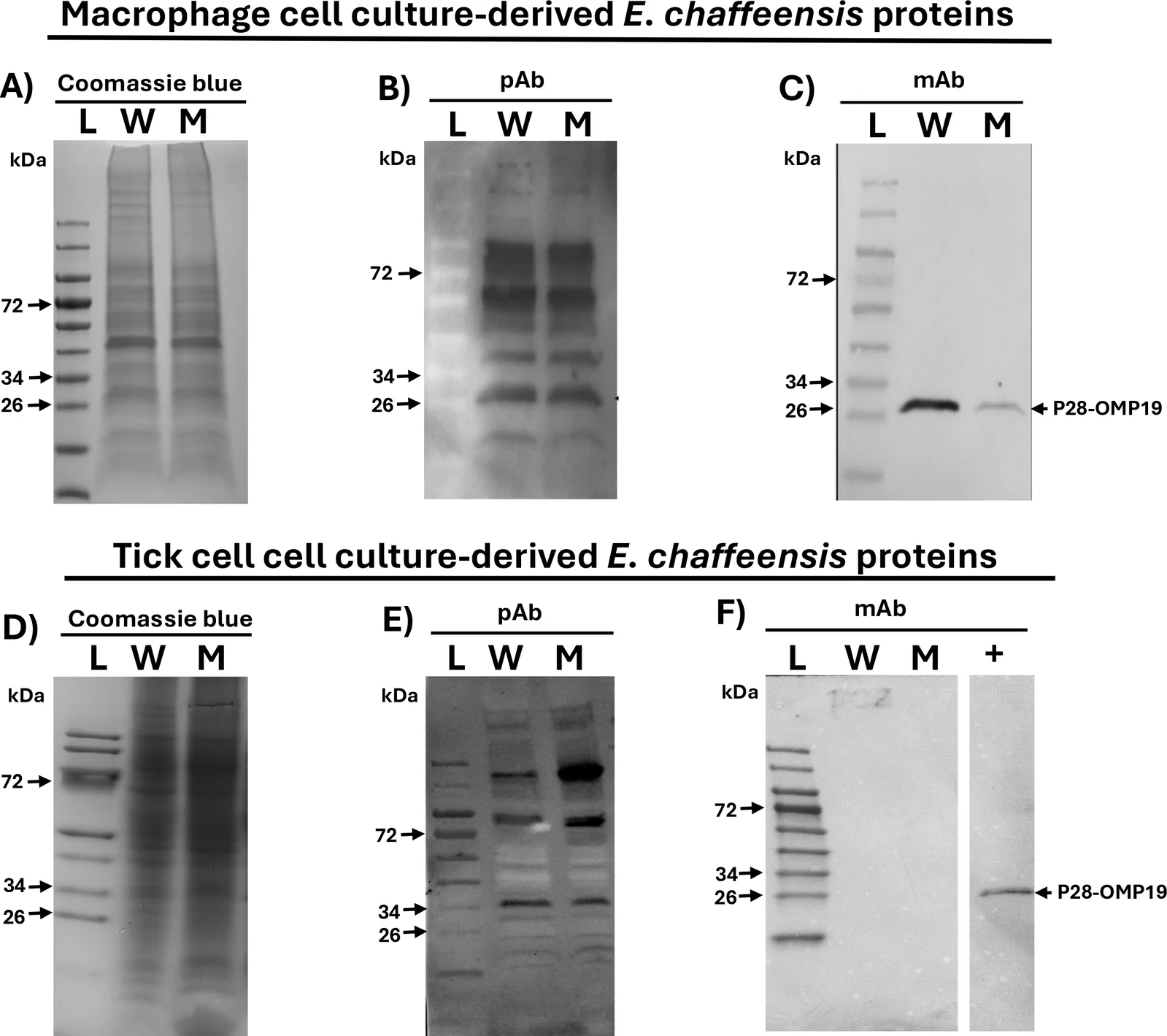
*E. chaffeensis ECH_1143* antisense mutation resulted in a reduction of p28-Omp19 protein expression in macrophage cultures assessed by Western blotting. Macrophage- and tick cell culture-derived *E. chaffeensis* wild-type and mutant lysates were subjected to PAGE and stained with Coomassie blue stain to visualize total proteins (**A and D**). Western blotting of the bacterial lysates assessed using *E. chaffeensis*-infected dog polyclonal sera (**B and E**) or with p28-Omp19-specific mAb (**C and F**).

**Fig 5:**
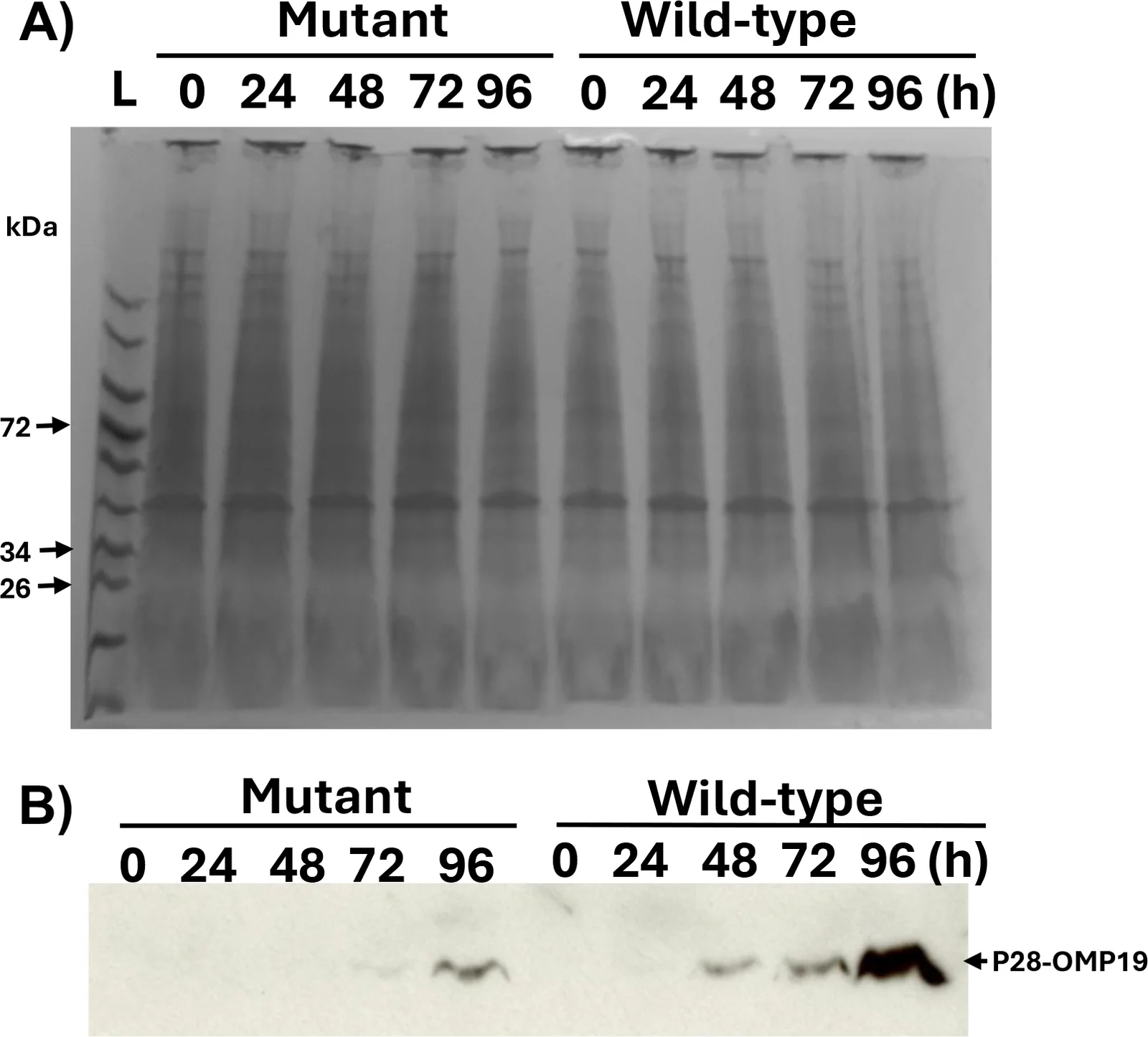
P28-Omp19 expression assessed over several days of growth of *ECH_1143* antisense mutant and wild-type *E. chaffeensis* cultured in the macrophage cell line. (**A**) Wild-type and mutant lysates were subjected to PAGE and stained with Coomassie blue. (**B**) Western blot analysis of lysates collected at different time points showed relatively higher p28-Omp19 expression over time in the wild-type compared with that in the antisense mutant.

Western blot analysis was performed to independently confirm the knockdown effect in the asRNA mutant on the p28-Omp19 protein expression. We used an equal amount of the proteins from the mutant and wild-type bacteria; total resolved protein profiles of the mutant and wild-type *E. chaffeensis* organisms were very similar in the Coomassie blue-stained gels, independent of growing in macrophage and tick cell cultures (Fig. 4A and D). Western blot analysis using *E. chaffeensis*-infected dog polyclonal sera (pAb) further supports the presence of equal amounts of resolved proteins, with no major notable differences in the total immunogenic proteins identified in the mutant compared to wild type (Fig. 4B and E). Whereas similar Western blot analysis using p28-Omp19-specific mAb 18.1 (Fig. 4C and F) revealed the presence of substantially less expressed protein for the mutant-derived protein fraction when cultured in the macrophage cell line. The p28-Omp19 protein expression was absent in the immunoblot containing tick cell culture-derived *E. chaffeensis* proteins independent of the proteins obtained from the mutant or wild type (Fig. 4F). We then assessed the changes in the protein expression profile over a period of 4 days in macrophage culture for the mutant and wild-type *E. chaffeensis*. We used equal amounts of proteins for each time-point assessment, which are evidenced by the resolved protein profiles for all the time points assessed for the mutant and wild type when assessed in the Coomassie blue-stained gel (Fig. 5A). The immunoblot analysis performed with the mAb 18.1 revealed a steady increase in the p28-Omp19 expression over time for the mutant and wild-type *E. chaffeensis,* with substantially less protein detected in the mutant-derived resolved protein fraction. The differences were more pronounced for the protein fractions recovered from 72 and 96 h time points (Fig. 5B).

### The asRNA mutant had attenuated growth in culture

**Fig 6:**
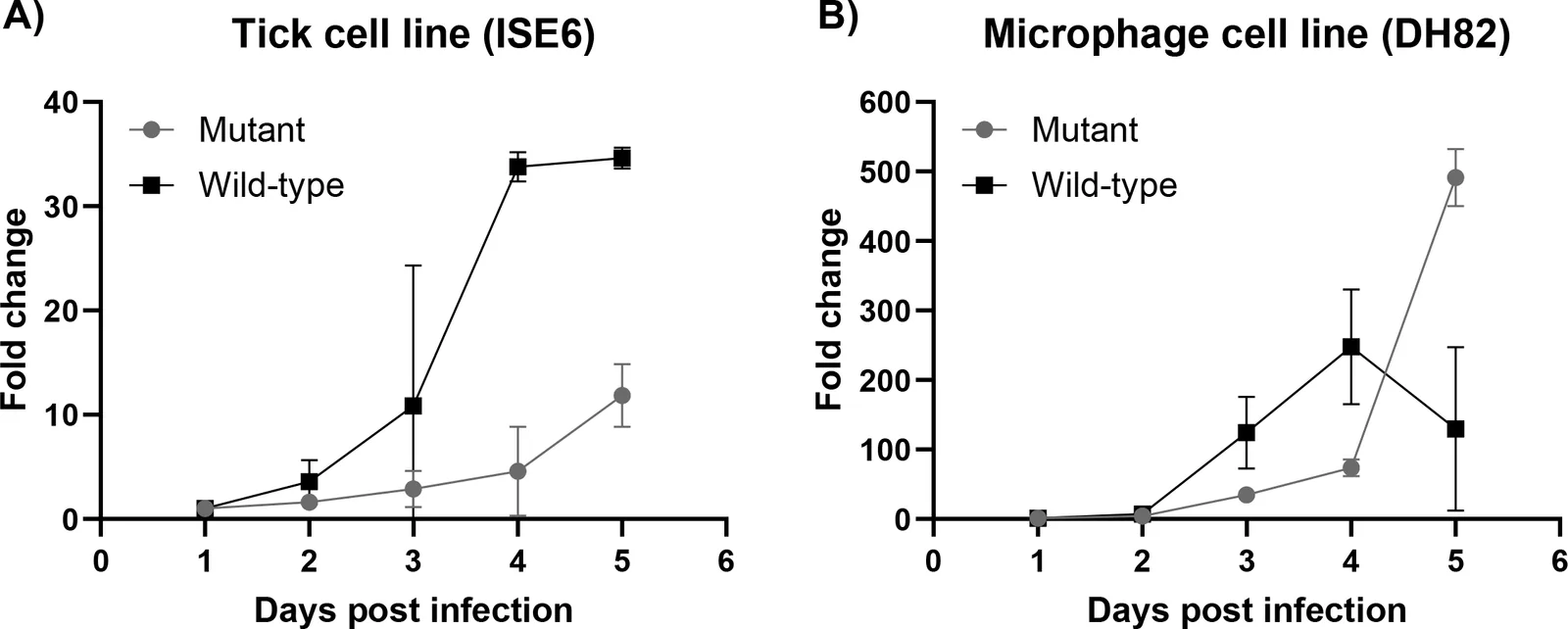
The growth kinetics of the antisense mutant in the macrophage and tick cell cultures. Growth of the mutant (gray line) was compared with the wild-type *E. chaffeensis* (black line) in tick cells (**A**) and macrophage cell line (**B**). Host cell-free bacteria were used to initiate infections, and bacterial growth was monitored for 5 days post-infection by quantifying *E. chaffeensis* 16S rRNA gene copy numbers by TaqMan-based real-time quantitative PCR assay. Bacterial numbers were presented as fold changes relative to day 1 post-infection. Each data point represents the mean of four replicates, and error bars indicate standard deviations. Statistical significance between the wild-type and antisense mutant at each time point was determined using multiple *t*-tests. In tick cells, the wild-type strain exhibited significantly greater growth compared to the mutant on day 4 (*P* = 0.045) and day 5 (*P* = 0.040), although overall retarded growth was evident in both cell types until day 4.

We then assessed the impact of the mutation on *E. chaffeensis* growth in the macrophage and tick cell cultures. Infection in cultures was initiated using host cell-free asRNA mutant and wild-type bacteria, and bacterial replication was monitored over time by quantitative PCR (qPCR) to determine bacterial genome copy numbers (Fig. 6). The antisense mutant displayed attenuated growth rates compared to the growth of the wild-type *E. chaffeensis* in both macrophage and tick cells. Significant decline in the bacterial growth was evident for the mutant throughout the assessment period in tick cell culture, with statistically significant differences observed on day 4 (*P* = 0.045) and day 5 (*P* = 0.040) post-infection relative to the wild type for the tick cell cultured bacteria (Fig. 6A). Similarly, the asRNA mutant displayed reduced growth kinetics compared with the wild type in the macrophage cell line, with substantially lower bacterial numbers than the wild type on days 3 and 4 (Fig. 6B). On day 5, the mutant’s growth was rapidly raised in the macrophage cell line.

### The asRNA mutation resulted in changes to the RNA expression from a paralog, p28-Omp14

**Fig 7:**
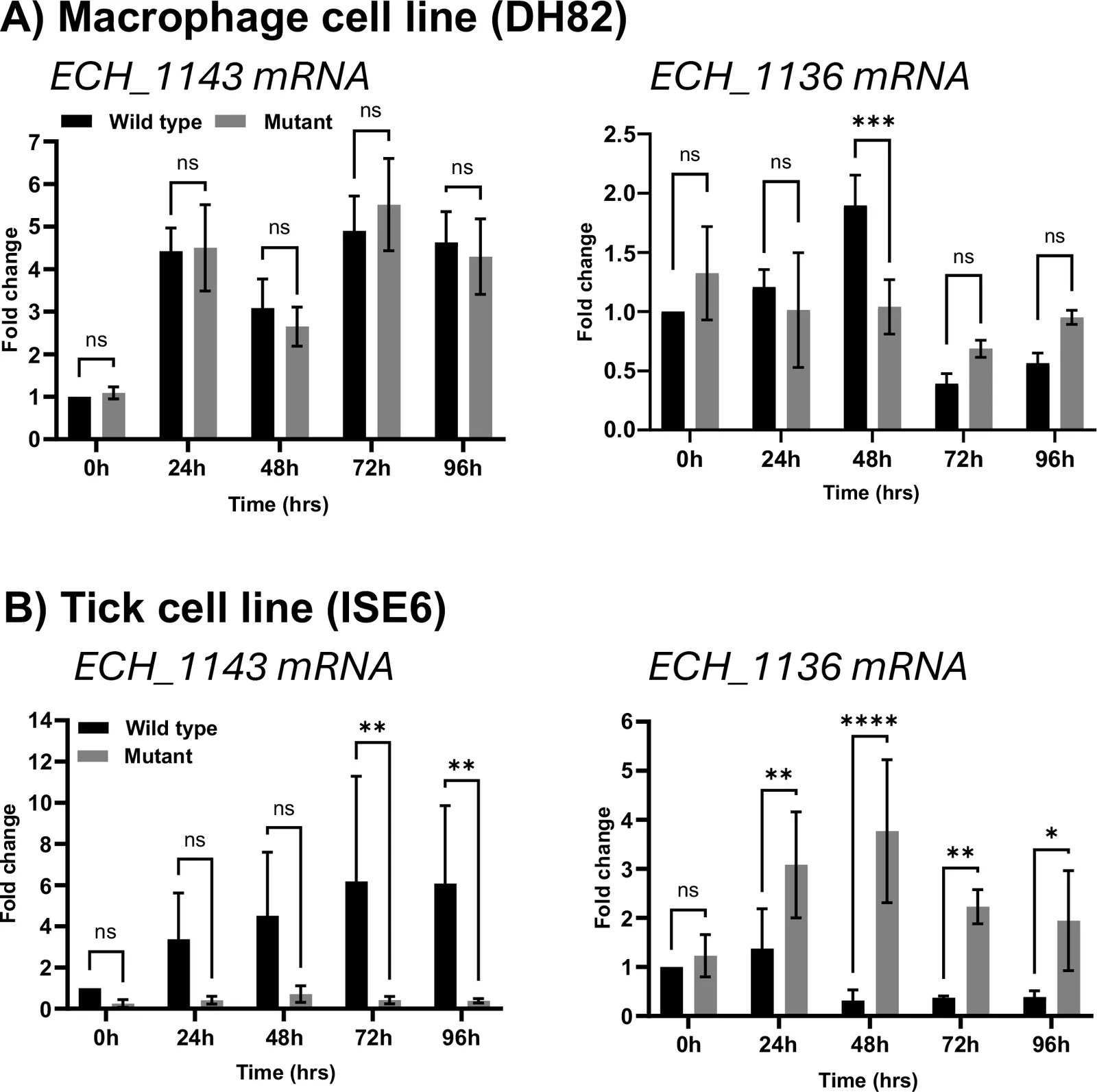
Transcription changes from genes *ECH_1143* and *ECH_1136* encoding the proteins p28-Omp19 and p28-Omp14, respectively, were assessed by qRT-PCR for the mutant and wild-type *E. chaffeensis* cultured in macrophage and tick cell cultures. Total RNA from synchronously cultured mutant and wild-type *E. chaffeensis* at different time points following infection from macrophage (**A**) and tick cells (**B**) was isolated. Real-time qRT-PCR analysis was performed to assess the changes in the mRNA levels from the two genes. Relative gene expression values are presented as fold changes compared to the wild type at 0 h post-infection. Data are shown as mean values ± standard deviations from three independent experiments, and each experiment was also performed in triplicate. Statistical significance was determined using a two-way analysis of variance (ANOVA), followed by Tukey’s multiple-comparison test (*P < 0.05, **P < 0.01, ***P < 0.001, and ****P < 0.0001).

Following confirming the knockdown of p28-Omp19 expression, we assessed if the reduction in the protein expression correlates with a decrease in transcript level from *ECH_1143*. We further examined how the asRNA mutation impacted transcription from a paralog gene, *ECH_1136*, encoding the p28-Omp14 protein. Protein and RNA expression from the two paralogs are known to differ *in vitro* and *in vivo*, with p28-Omp19 expression being higher when the pathogen replicates in macrophages but not in tick cells, while the protein synthesized from *ECH_1136* (p28-Omp14) is primarily detected in *E. chaffeensis* grown in tick cells (29, 30, 34). RNA expression, assessed by qRT-PCR, revealed very similar transcript levels for the *ECH_1143* transcript for the mutant and wild-type in the macrophage culture, although time-dependent changes in the levels of transcription were evident (Fig. 7A, left panel). Similarly, transcript levels did not significantly differ for *ECH_1136* in macrophage cultures, except for the 48-h time point, where expression was significantly decreased in the mutant compared to wild-type *E. chaffeensis* (Fig. 7A, right panel). Similar assessment in tick cell cultures revealed a notable reduction in the mRNA levels of *ECH_1143* for the mutant, with a significant decrease in transcript levels observed for the later time points (72 and 96 h) (Fig. 7B, left panel). The mRNA levels for *ECH_1136* for the mutant were significantly higher for all time points of assessment compared to wild type (Fig. 7B, right panel). In addition, we assessed *ECH_1143* sense mRNA transcript levels at 72 h post-infection by RT-PCR in the asRNA mutant and wild-type bacteria grown in macrophage and tick cell cultures (Fig. S1). Comparable *ECH_1143* mRNA levels were observed between the mutant and wild-type for the RNA recovered from both cell backgrounds, whereas *ECH_1143* transcript abundance was reduced in the mutant relative to the wild type when cultured in the tick cell line. These observations are consistent with the qRT-PCR data (Fig. 7).

### Ribonuclease III (RNase III) expression in *E. chaffeensis* is significantly higher during its growth in tick cells

**Fig 8:**
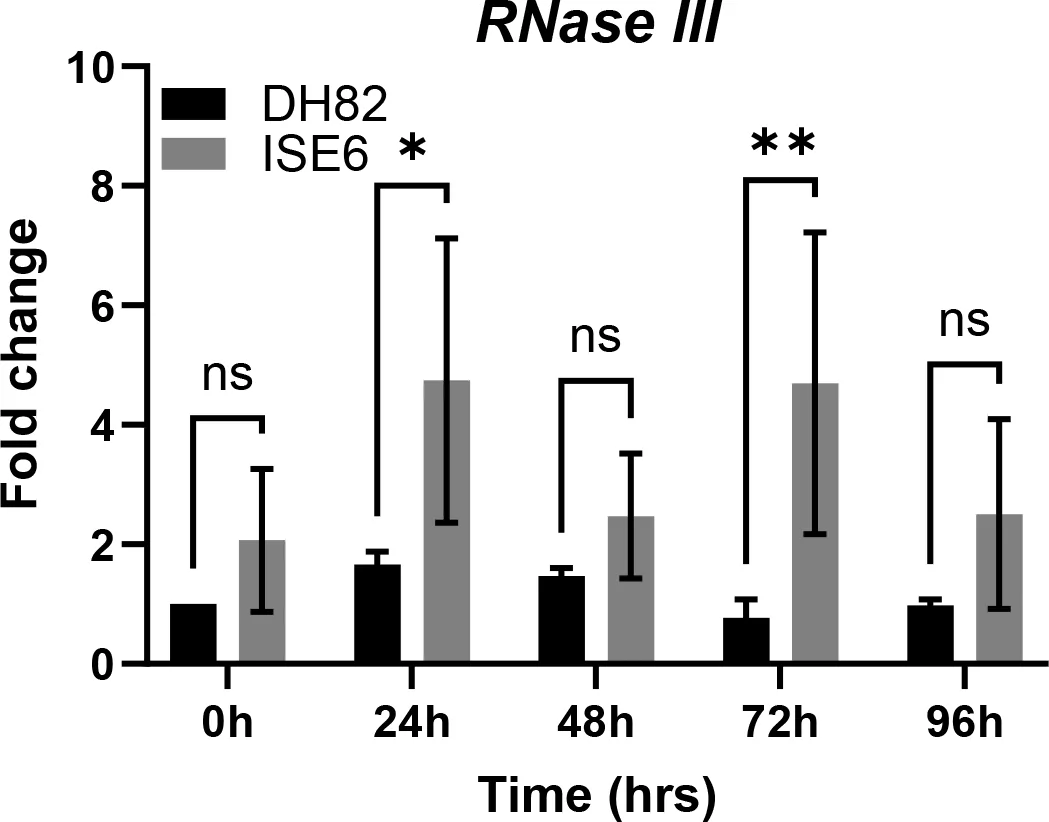
The *RNase III* transcript levels encoded from *ECH_1054* for wild-type *E. chaffeensis* grown in the macrophage and tick cells were measured. Total RNA isolated from synchronously cultured wild-type *E. chaffeensis* at different time points in culture. Real-time qRT-PCR analysis was performed to assess the expression levels of *RNase III* transcripts. Expression values are presented as fold changes relative to the 0 h time point. Data are shown as mean values ± standard deviations from three independent experiments, each performed in triplicate. Statistical significance was determined using a two-way analysis of variance (ANOVA) followed by Tukey’s multiple-comparison test (*P < 0.05 and **P < 0.01).

We predicted that the observed reduction in p28-Omp19 expression as a result of the asRNA mutation correlates to reduction in *ECH_1143* mRNA when cultured in macrophages. One of the mechanisms of reduction in protein expression in bacteria with sense and antisense RNA duplex formation is to sterically block translation initiation without degrading the duplex (35–37). This can occur when RNase III expression is lower (38). Bacterial RNase III regulates gene expression and mRNA turnover by degrading double-stranded RNA (dsRNA) molecules (39, 40). We, therefore, evaluated if RNase III expression levels differ in wild-type *E. chaffeensis* when cultured in macrophage and tick cell lines (Fig. 8). We detected higher *RNase III* transcript levels for the bacteria in tick cell cultures throughout the 4-day assessment period compared to macrophage culture, with significant differences that were evident at 24 and 72 h time points. The differences in RNase III expression may contribute to altered processing of asRNA mRNA hybrids when the mutant *E. chaffeensis* is cultured in tick cells, with pronounced degradation and less obvious degradation in the macrophage cultures.

## DISCUSSION

In the current study, we developed a novel targeted mutagenesis method in *E. chaffeensis* enabling the generation of *trans*-acting asRNA. The novelty of the method stems from expressing asRNA driven by the duplicated gene promoter involved in transcribing the sense mRNA, thus providing simultaneous expression of asRNA with sense mRNA, leading to an effective means to downregulate the targeted gene expression. We aimed to express the antisense RNA at the same time a sense RNA is made and from a distant genome location. Therefore, we engineered the asRNA mutation at a distant genomic mutation landing site (3′ end of *ECH_0230*). Because bacterial organisms lack a nucleus, ribosomes act on the bacterial mRNAs as they progress to protein translation while the mRNA is still being actively transcribed by RNA polymerase; thus, transcription and translation are coupled in bacteria. With this, we reasoned that there would be a short delay in the asRNA made near *ECH_0230* traveling to the sense RNA for initiating the knockdown effect, thus allowing partial protein synthesis to occur. Prior substantial research demonstrates that asRNA rarely achieves 100% efficiency, as it heavily depends on a target location, cellular environment, and the molecular mechanism involved (41, 42). Most highly efficient knockdowns typically involve blocking the ribosomal binding site, while our method is targeted only to the protein-coding region of the mRNA starting from the initiation codon. Because our designed mutation is distantly located relative to *ECH_1143*, we also reasoned that we would only have a partial knockdown. The data presented in this manuscript were consistent with our hypothesis. When designing the mutational construct, we ensured that the mutation did not alter the integrity of the sequences at a distal genomic site where the mutation was introduced and also confirmed by whole-genome sequence analysis that the mutation was present only at the intended genomic location and that there were no off-target insertions detected. This is the first description of an antisense knockdown system for a rickettsial pathogen, and we believe that the method will be broadly applicable for other bacterial pathogens. We demonstrated the proof of concept in *E. chaffeensis* for generating asRNA expression by means of allelic exchange mutagenesis.

Noncoding RNAs regulate gene expression in a wide range of species (43–46). In bacteria, such RNAs encoded for asRNAs modulate protein expression post-transcriptionally by base-pairing interactions with target sense mRNAs (46, 47). Noncoding antisense oligonucleotide (ASO)-mediated knockdown of gene expression is a research tool aiding investigations in various organisms (20, 48). However, such studies are yet to be described in any known rickettsial pathogens. Noncoding RNAs, including *cis*-acting and *trans-*acting RNAs, naturally exist in *Rickettsia* species and may regulate gene expression (49–52). The existence of noncoding asRNAs is also reported in *Anaplasma phagocytophilum* and *Wolbachia pipientis* (53, 54), while their functional roles remain unclear. An ASO-based gene knockdown relies on the use of a delivery system tailored to an organism under investigation (20, 21, 55). In a plasmid-based delivery system, antisense DNA sequences are cloned downstream of an unrelated promoter to allow transcription of asRNA following such plasmid’s introduction into bacteria, which results in the translation knockdown with or without promoting the sense RNA degradation (23, 56, 57). Traditional bacteria mutagenesis methods are particularly challenging to use for obligate intracellular bacteria, including all known rickettsial pathogens. Their dependence on the host cell for survival/replication makes working with the bacteria outside the host environment rather exceptionally challenging and delicate. Additionally, the absence of natural plasmids harbored within the genomes of Anaplasmataceae family pathogens, including *E. chaffeensis*, makes plasmid-based studies refractory (8). Similarly, inducible promoter-based systems are yet to be developed for supporting mutational studies in Anaplasmataceae pathogens. Transfection of chemically synthesized short antisense oligonucleotides may be used as an alternate tool for knocking down gene expression (20, 58). This is also not a practical option, as such oligonucleotides are highly prone to nuclease degradation. Recent studies described the application of transfected stable synthetic polymers, with characteristics similar to RNA and DNA, known as peptide nucleic acids (PNAs), to inhibit gene expression in *E. chaffeensis* (59). While this is a valuable alternative, the method is limited to transiently reducing protein expression (60).

The genetic modification method reported in this study involved creating a mutation at a distal genomic site to express asRNA complementary to the partial coding mRNA from *ECH_1143*. In this approach, we engineered the mutation downstream of the *ECH_0230* coding sequence, a region previously demonstrated as amendable for mutation (8). Further, we carefully designed the mutational construct to ensure that the integrity of the genomic sequences at the mutation site is not altered; the mutational design included duplicating a segment of DNA 3′ to the *ECH_0230* coding region to ensure that we do not delete any noncoding and regulatory sequences involved in the transcription from it or the gene positioned 3′ to it (*ECH_0232*). We further confirmed that this insertion mutation did not alter RNA expression from a gene located upstream and downstream from the insertion site. The mutational construct design included using the entire noncoding DNA segment located downstream of *ECH_1142*, with a predicted transcription terminator sequence, and upstream of *ECH_1143*, which we previously reported as having a well-defined functional promoter (61, 62). The construct then included the insertion of the 209-base-pair open reading frame sequence from its initiation codon, engineered in the reverse and complementary orientation. Our engineered mutagenesis cassette included the promoter segment of the target gene (*ECH_1143*) for facilitating the asRNA expression concurrently with the sense RNA synthesis. Synchronous expression of sense and antisense mRNAs is expected to result in an effective reduction in the protein output. The data presented in the current study confirm the protein expression knockdown, evidenced by two independent methods: confocal microscopy and Western blot analysis.

Protein expression from *ECH_1143* is host cell-specific, both *in vitro* and *in vivo* (29, 30, 34). Prior studies demonstrated that it is a highly expressed immunogenic protein in *E. chaffeensis* during its growth in macrophage cultures, while it is not known to be expressed during its replication in a tick cell culture. *ECH_1136* is one of the 22 gene paralogs, including *ECH_1143*, existing in the *E. chaffeensis* genome, and its encoded p28-Omp14 protein is the only known expressed protein during bacterial replication in the tick cell environment (29, 34), although transcripts for both genes are known to be expressed in both vertebrate and tick cell cultures (34, 63). Similarly, the host-specific differential protein expression from *ECH_1143* and *ECH_1136* is documented *in vivo*; both proteins are known to be expressed during infection of a vertebrate host, while p28-Omp14 protein is the only known expressed protein in *E. chaffeensis*-infected ticks (29). The data presented in the current study demonstrate that the novel antisense mutation caused a substantial reduction in protein expression of *ECH_1143* when the pathogen was cultured in a macrophage cell line. Furthermore, we also demonstrated that protein expression was undetectable for it during replication in tick cells, independent of wild-type or mutated *E. chaffeensis*, despite the presence of mRNA. These data agree with prior reports of the absence of p28-Omp19 expression in tick cell cultures and ticks, despite the presence of transcripts (29, 34, 63).

We observed the presence of the *ECH_1143* transcript in tick cells for wild type, despite no protein expression, and the asRNA mutation reduced the transcript levels, with increased transcription from a paralog, *ECH_1136*. Whereas the transcript levels remained similar for the wild type and mutant in the macrophage culture for *ECH_1143*, and similarly for *ECH_1136*, except for the 48 h time point assessment. Interestingly, the mutation caused a fitness defect for *E. chaffeensis* growth in both macrophage and tick cell backgrounds. The data suggest that the antisense mutation knockdown of mRNA in tick cells may have a complementary effect in enhancing the transcription of *ECH_1136*. It is unclear why p28-Omp19 protein expression was not evident for the pathogen during its growth in tick cells, despite the presence of its transcript, which was consistent with prior published work (34, 63). One possible explanation is that the bacterium may have different translational regulation, with no protein expression or too low to be detected by the methods used in identifying the protein expression: confocal microscopy and Western blot methods. While warranting additional investigations, the data also suggest that bacterial gene regulation is heavily impacted by its replication in vertebrate and invertebrate host cell backgrounds. Indeed, prior published research reported host-specific differences in *E. chaffeensis* gene and protein expressions (29, 34, 63). The lack of RNA expression changes in the mutant compared with wild type, while causing the protein expression knockdown, suggests that asRNA only blocked translation but not mRNA degradation in macrophage cultures. Conversely, *ECH_1143* mRNA expression reduction was evident for the mutant in tick cells, and the data suggest that asRNA induced sense mRNA breakdown during growth in tick cell cultures. Binding of asRNA to target mRNA blocks translation by inhibiting ribosome access; alternatively, it can promote RNase III-mediated degradation of the duplex, and both mechanisms lead to the protein synthesis reduction (64). Therefore, the interactions between sense RNA and asRNA do not always lead to mRNA degradation (37). Instead, the RNA duplex may create a specific processing site, which can result in a translationally inactive mRNA or produce a mature and stabilized form of duplex mRNA (37). The asRNA of *ECH_1143* when bound within a window of the first 70 codons from the start codon may have likely caused decreased p28-Omp19 translation without inducing degradation of the sense RNA during the mutant’s replication in the macrophage cell line.

In *E. coli*, RNase III mediates the breakdown of sense-antisense hybrids (65, 66). Our results demonstrate that the *E. chaffeensis RNase III* transcription differed for the pathogen when grown in macrophage and tick cell cultures. RNase III is significantly higher during *E. chaffeensis* replication in tick cell cultures compared to its replication in macrophage cultures. These data were similar to the previously reported genome-wide *E. chaffeensis* gene expression assessment, which also suggests that *RNase III* mRNA levels are lower for the organisms cultured in human monocytic cells (THP-1) compared to tick cells (ISE6) (67). Thus, it is likely that having higher RNase III expression in tick cell cultured organisms may have promoted the breakdown of sense and antisense mRNA duplexes. This is on the assumption that the RNase III transcription mirrors protein translation. In particular, the RNase III transcription changes may not necessarily reflect RNase III protein changes and thus require further validation by protein analysis. The data also suggest that *E. chaffeensis* employs distinct post-transcriptional regulatory mechanisms in macrophage and tick cell environments.

In summary, the novel mutational approach is established for the first time for an obligate intracellular bacterial pathogen, *E. chaffeensis*. It is a pertinent alternative for investigating genes that are likely essential for pathogens having reduced genomes. The method is unique in allowing simultaneous asRNA expression, as both the sense and antisense RNAs are transcribed from the same gene promoter, as the mutation included the duplicated gene promoter that belongs to the same gene. We anticipate that this innovative molecular tool will greatly facilitate research in investigating essential pathogen genes and is likely applicable to other pathogens having reduced genomes, such as those within the order of Rickettsiales and possibly for other important pathogenic microorganisms belonging to the genera *Borrelia*, *Chlamydia*, and *Coxiella*.

## MATERIALS AND METHODS

### *In vitro* cultivation of *E. chaffeensis*

*E. chaffeensis* Arkansas isolate and the antisense RNA mutant were continuously cultivated in the *Ixodes scapularis* embryonic cell line (ISE6) and in the canine macrophage cell line (DH82) as previously described (46).

### Construction of homologous recombination plasmids and segments

The primers designed for use in generating the recombinant plasmid are listed in Table S1. All PCR experiments for preparing the recombinant plasmid construct were performed using Q5 High-Fidelity DNA Polymerase (New England Biolabs, Ipswich, MA, USA). The 5′ *E. chaffeensis* left homology arm (L) with genome coordinates of the amplified segment spanning from 218,537 to 219,618 and 3′ right homology arm (R) with genome coordinates of the segment are 219,083 to 220,133 were amplified. The L segment spans part of the *ECH_0230* open reading frame (ORF) and the entire noncoding region located 3′ to the ORF, and the R segment also contains the entire noncoding region located 3′ to the *ECH_0230* ORF and the partial ORF belonging to *ECH_0232* (3′ portion of the ORF) (GenBank accession no. CP000236). The complete noncoding DNA segment located upstream to *ECH_1143* and downstream to *ECH_1142,* with well-defined functional promoter elements (61, 62) (genome coordinates: 1,163,653–1,163,967) (here referred to as the *ECH_1143* promoter segment), and the first 209 base pair (bp) open reading frame sequence of *ECH_1143* (genome coordinates: 1,164,176–1,163,968) were independently amplified using wild-type *E. chaffeensis* genomic DNA as the template. The *ECH_1143* promoter segment and the 209 bp *ECH_1143* ORF were cloned in reverse orientation into a plasmid; the assembled fragment was referred to as AS. The *tuf* promoter (T), along with the mCherry (M) and gentamicin resistance protein coding sequence (G), were obtained from the AM581-KO-tuf-mCherry-Gent plasmid (10). All these fragments were cloned into the pGGA plasmid (New England Biolabs) in the following order according to the manufacturer’s Golden Gate Assembly kit protocol (New England Biolabs): L, T, M, G, AS, and R. Recombinant plasmid was transformed into *E. coli* DH5α, and plasmid DNA was recovered. Correct assembly of the recombinant plasmid DNA segments was confirmed by Sanger sequencing with T7 and SP6 primers (Integrated DNA Technologies, Coralville, IA, USA), which anneal upstream and downstream of the recombinant DNA segment in the pGGA plasmid. The final construct was named pHR-*ECH_1143*-Antisense (Fig. 1). This plasmid served as the template for amplification of the allelic exchange fragment containing the recombinant segment spanning from L to R in the plasmid. PCR products were then purified with the QIAquick PCR Purification Kit (Qiagen, Germantown, MD, USA) prior to use for the mutation generation.

### Electroporation of *E. chaffeensis* with the linear DNA fragments and the selection of the mutant

Host cell-free *E. chaffeensis* organisms were recovered as previously described i 11). The linear purified DNA fragments (3 µg) generated above from the pHR-*ECH_1143-*Antisense plasmid were added to the host cell-free *E. chaffeensis* organisms in 45 µL volume, mixed gently, and transferred to a 1 mm electroporation cuvette (Bio-Rad Laboratories, Hercules, CA, USA), placed on ice for 15 min prior to subjecting to electroporation at 2,000 volts, 25 µF, and 400 Ω setting (Gene Pulser Xcell, Bio-Rad Laboratories). The electroporated cells were transferred to a microcentrifuge tube containing 0.5 mL of stock FBS and 1 mL of uninfected ~1 × 10^6^ ISE6 cell suspension in the tick cell culture infection medium, then centrifuged at 5,000 × *g* for 5 min, and incubated at room temperature for 15 min. The cell suspension was transferred to 5 mL culture medium, transferred to a T25 flask with a confluent ISE6 cell monolayer, and incubated for 24 h at 34°C in a humidified incubator. Subsequently, 80 µg/mL of gentamicin was added to the culture medium after 24 h, and cultures were monitored for several weeks until mCherry-expressing *E. chaffeensis* infection was detected and the wild type was not detected.

### Confirming the presence of *E. chaffeensis* mutants

The *E. chaffeensis* culture resistant to the presence of gentamicin in medium was screened to confirm the mutation. Genomic DNA recovered from the mutant cultures was used to perform two different PCR assays. PCR I was targeted to define the clonal purity of the mutant, where the primers used for the assay were targeted to the genomic regions upstream and downstream of the allelic exchange insertion site, which should only yield the predicted larger-size fragment for the mutant if clonally pure. PCR II was targeted to the genomic region 5′ to the allelic exchange site and to the insertion-specific DNA; PCR primers for both PCR I and II were listed in the Table S1. The PCR products were resolved on a 0.9% agarose gel to check for the presence of specific predicted amplicons. Additionally, whole-genome sequencing was performed using mutant genomic DNA on Nanopore sequencing platform (Plasmidsaurus Inc., KY, USA) to independently confirm the mutation and to rule out any off-target insertions. To remove Nanopore adapters and to perform quality filtering, Porechop v.024 (68) and Filtlong v.0.2.1 (https://github.com/rrwick/Filtlong) were used with default settings. The remaining high-quality reads were mapped to the *E. chaffeensis* isolate reference genome (CP000236) and the insert construct (mCherry gene, gentamicin gene, the promoter of *ECH_1143*, and antisense sequence of *ECH_1143*) using Minimap2 v.2.28 (69). Mapped reads were extracted via samtools v.1.21 (70) and assembled *de novo* using Flye v.2.9.6 (71). The Artemis genome browser and annotation tool are used to visualize the sequence data (72).

### Preparation of RNA samples

For synchronized infection experiments, macrophages and tick cells were seeded into six-well plates at a density of 1 × 10^5^ cells per milliliter and cultured until approximately 70–80% confluence was reached. Each well was then inoculated with host cell-free wild-type or mutant *E. chaffeensis*. Following incubation for 2–3 h to allow bacterial internalization, the inoculum was removed and replaced with fresh culture medium. This time point was designated as 0 h post-infection (hpi). Cells from one well were harvested immediately (0 hpi) and subsequently at 24-h intervals for four consecutive days. Total RNA was isolated from harvested cells using TRI Reagent (Sigma-Aldrich, St. Louis, MO, USA) according to the manufacturer’s instructions. Genomic DNA contamination was removed by treatment with RQ1 RNase-Free DNase (Promega, Madison, WI, USA) prior to downstream analyses.

### Detection of antisense RNA and sense mRNA expression by RT-PCR

To assess the expression of the engineered antisense RNA (asRNA) and the endogenous *ECH_1143* sense mRNA, RNA samples were normalized based on 16S rRNA expression and then used for first-strand cDNA synthesis with the SuperScript III First-Strand Synthesis System for RT-PCR (Invitrogen, Carlsbad, CA, USA). The resulting cDNAs served as templates for subsequent PCR analysis. Primers designed for detection of the sense mRNA and asRNA transcripts were carefully designed and were listed in Table S1. The primer P4 was designed to anneal downstream of the asRNA sequence, which was expected to be present only in the asRNA mutant RNA or its genomic DNA, as it was engineered to anneal within the inserted 3′ end intergenic segment of *ECH_0230,* which was part of the insertion design. The resulting cDNA was used as the template for PCR amplification with primer pair P3 and P4, yielding the expected amplicon of 263 bp. The design allows the sense RNA to be positive for both wild-type and mutant, whereas the asRNA transcript was expected to be positive only in the mutant. PCR amplification was performed for sense RNA expression using 1 μL of cDNA template and GoTaq Green DNA Polymerase according to the manufacturer’s instructions (Promega, Madison, WI, USA). For the asRNA, the assay was carried out using 1 μL of cDNA template and Q5 Hot Start High-Fidelity DNA Polymerase (New England Biolabs) following the manufacturer’s protocol. PCR products were resolved by electrophoresis in a 2% agarose gel and stained with GelRed (Biotium, Fremont, CA, USA) and visualized using a UV transilluminator.

### Assessment of 5′ and 3′ flanking gene’s expression by RT-PCR

To determine whether insertion of the asRNA construct affected transcription of neighboring genes, the RNA expression levels of one gene each upstream and downstream of the insertion site, *ECH_0230* and *ECH_0232*, respectively, were evaluated by reverse transcription-PCR (RT-PCR). RNA samples harvested at 72 h post-infection, as described above, were normalized based on 16S rRNA expression and used as templates for semi-quantitative RT-PCR analysis using the SuperScript III One-Step RT-PCR System with Platinum Taq DNA Polymerase (Thermo Fisher Scientific, Waltham, MA, USA). Gene-specific primers for *ECH_0230* and *ECH_0232* were listed in Table S1. The amplified products were resolved on a 1.5% agarose gel and stained with GelRed (Biotium) and visualized using a UV transilluminator.

### P28-Omp protein expression in the mutant and wild-type *E. chaffeensis* assessed by confocal microscopy analysis

At approximately 80% infection*, E. chaffeensis* wild-type and mutant-infected macrophage and tick cell cultures were seeded in a 24-well plate (containing a glass slip) or four-chambered slides at a density of 1 × 10^5^ cells per well, respectively. The supernatant medium was removed, and the cells were washed three times with phosphate-buffered saline (PBS). Cells were fixed with 4% paraformaldehyde solution for 10 min at room temperature. Cells were then permeabilized with 0.2% Triton X-100 in PBS (PBST) for 15 min at room temperature, then washed three times with PBS. Cells were blocked with 5% bovine serum albumin (BSA) for 2 h at room temperature, then incubated with the primary antibody, p28-Omp19-specific monoclonal antibody, mAb 18.1 raised in mice (1:400) (33), at 4°C overnight. Cells were washed three times with 1× PBST. Cells were then incubated with Alexa Fluor 488-conjugated Goat Anti-mouse IgG (H + L) antibody (1:2000) for 2 h at room temperature with cells protected from light. After three washes with PBST, cells were stained with DAPI solution for 10 min at room temperature (Thermo Fisher Scientific) to stain nucleus or morula containing *E. chaffeensis* organisms. Images were captured using an LSM 880NLO laser (Carl Zeiss, Germany) and MP confocal microscopes for the detection of mutant and wild-type bacteria for the presence of inclusions with or without the expression of mCherry and for the specific expression of p28-Omp 19, and the images were further processed using ImageJ software.

### Protein expression over time in *E. chaffeensis* wild type and mutant when cultured in the macrophage or tick cell lines and assessed by Western blot analysis

Macrophage- and tick cell culture-derived *E. chaffeensis* wild type and mutant were purified as previously described (34). Bacterial lysates were prepared, and total protein concentrations were measured using the bicinchoninic acid (BCA) protein assay as per the manufacturer’s recommendations (Thermo Fisher Scientific). Lysates were then subjected to sodium dodecyl sulfate polyacrylamide gel electrophoresis (SDS-PAGE) using equal amounts of proteins and then either stained with Coomassie blue stain to visualize the separated proteins or transferred to a polyvinylidene difluoride (PVDF) membrane and subjected to Western blot analysis. For the blot analysis, the membranes containing the transferred proteins were blocked with 5% non-fat dry milk in Tris-buffered saline with 0.1% Tween 20 (TBST) for 1 h at room temperature, then incubated with mAb 18.1 (1:1,000) specific to detecting *E. chaffeensis* p28-Omp19 or polyclonal sera from an *E. chaffeensis*-infected dog (1:500) (73, 74) at 4°C overnight. After three washes with TBST for 5 min each, membranes were incubated with horseradish peroxidase (HRP)–conjugated goat anti-mouse or anti-dog at a 1:4,000 dilution in TBST for 1 h at room temperature. Protein bands were detected using an enhanced chemiluminescence (ECL) Western blot detection kit (Thermo Fisher Scientific) according to the manufacturer’s instructions. The images of Western blots were captured using iBright Imaging Systems (Thermo Fisher Scientific).

### Growth assessment of the mutant in macrophage and tick cell cultures by TaqMan probe-based quantitative PCR

To evaluate the effect of *ECH_1143* asRNA expression on *E. chaffeensis* growth fitness, the replication kinetics of the mutant and wild type were cultured in macrophage and tick cell lines and assessed by real-time quantitative PCR. Host cell-free bacteria were first prepared from approximately 90% infected 75 cm² culture flasks containing either the antisense mutant or wild-type *E. chaffeensis* grown in DH82 or ISE6 cells, respectively. Briefly, infected cells were disrupted by vortexing with glass beads using BeadBug 3-Position Microtube Homogenizer (Benchmark Scientific, Sayreville, NJ, USA), and unbroken host cells and large cellular debris were removed by centrifugation at 1,000 × *g* for 10 min. The resulting supernatant containing cell-free bacteria was passed through a 2 μm filter. The purified bacteria were then used to infect confluent monolayers of the macrophage or tick cells, respectively, which were maintained in 75 cm² flasks. Following a 2-h incubation to allow bacterial host cell entry, the monolayers were washed twice with cold Dulbecco’s phosphate-buffered saline (DPBS) (Thermo Fisher Scientific) to remove any unbound extracellular bacteria, and final cultures in the flasks were resuspended with 25 mL of fresh culture medium and distributed equally (5 mL) into five 25 cm² flasks. Cultures were incubated at 37°C for the macrophage cell line and 34°C for tick cells, and cultures were maintained for 1–5 days. At designated time points (days 1–5), infected cultures from each flask were harvested, and total DNA was isolated for bacterial growth assessment. Bacterial burden was quantified by real-time quantitative PCR (qPCR) targeting the *E. chaffeensis* 16S rRNA gene, as we described previously (9). Bacterial numbers were calculated based on 16S rRNA gene copy numbers and expressed relative to the initial inocula detected on day 1 post-infection. The experiment was performed twice with two replicates each time (*n* = 4).

### RNA analysis by TaqMan probe-based real-time quantitative RT-PCR (qRT-PCR) to define the impact of the antisense mutation

RNA samples prepared, as described above, were equalized relative to 16S rRNA expression as we described earlier in reference 75. Primers and probes for the TaqMan probe-based RT-PCR assays for determining the RNA expression from *ECH_1136*, *ECH_1143*, and the *RNase III* gene (*ECH_1054*) were listed in the Table S1, which were used to evaluate changes in the mRNA levels for each gene for all harvested RNAs. The fold differences relative to the zero time point were calculated by 2^−ΔΔC*t*^ method (76). The analysis was performed three independent times and represented the mean values of the three experiments.

### Statistical analysis

Statistical analyses were carried out using GraphPad Prism 12 (GraphPad Software, San Diego, CA, USA). Differences between groups were evaluated using two-way analysis of variance (ANOVA) or multiple *t*-tests, as appropriate. Data are presented as the mean ± standard deviation (SD). Differences were considered statistically significant at *P < 0.05 and highly significant at *P < 0.01.

## SUPPLEMENTAL MATERIAL

Supplemental material jb.00334-26-s0001.pdf Table S1 and Fig. S1.

ASM does not own the copyrights to Supplemental Material that may be linked to, or accessed through, an article. The authors have granted ASM a non-exclusive, world-wide license to publish the Supplemental Material files. Please contact the corresponding author directly for reuse.
