# Supplementary material for: A targeted mutational strategy aiding in generating antisense RNA to knockdown the Ehrlichia chaffeensis P28-outer membrane protein 19 expression
Source: J Bacteriol. 2026 Aug 4;208(8):e00334-26. doi: 10.1128/jb.00334-26 (PMC13492146; doi:10.1128/jb.00334-26)
Supplement: Supplementary file 1 [file jb.00334-26-s0001.pdf]

Table S1: Oligonucleotides use in this study

| Name | Sequences (5'----3') | Orientation | Use |
|---|---|---|---|
| **Construct pHR-ECH_1143-Antisense plasmid** | | | |
| RG147 | _CAAGTACAGCAACATAAACTAGTAGT CTTTTAATTTTATTATTGCCACATG | **Forward** | p28-Omp19 promoter |
| RG150 | ATATAACCTAATAGTGACAAATAAAT | **Reverse** | |
| RG148 | TTATTTGTCACTATTAGGTTATAT TTTTGCTTCAGTCCAAAC | **Forward** | p28-Omp19 Antisense |
| RG149 | ATATATAAACTGTAGTACC ATGTGATGAATTACAAAAAAGTTTTCATAAC | **Reverse** | |
| RG897 | CTGTTGGGTCTCCTGAC TCT TTTAATTTTATTATTGCCACATG | **Forward** | p28-Omp19 promoter-Antisense |
| RG898 | CTGTTGGGTCTCC TGTG ATGAATTACAAAAAAGTTTTCATAACAA | **Reverse** | |
| RG895 | CTGTTGGGTCTCGGGAGGATGGATGGGTTACTTCATTTT | **Forward** | LHA |
| RG896 | CTGTTGGGTCTCG ATAC TCTGACATACCTATCTTAGAAT | **Reverse** | |
| RG899 | CTGTTGGGTCTCC CACA TGGTACTACAGTTATATATAATCA | **Forward** | RHA |
| RG900 | CTGTTGGGTCTCCATGGAAGACACACAAGAACATGAC | **Reverse** | |
| RG845 | TTGGGTGGTCTCGGTCATTTTATGTTGCTGTACTTGGATC | **Forward** | tuf-mcherry-genta |
| RG846 | TTGGGTGGTCTCGGTATAAAAATGTGACTATTAATTTTGACTTTT | **Reverse** | |
| **For producing the linear template of homologous recombination** | | | |
| RG26 | GCTTTGGATTGTTTGTCTTA | **Forward** | Linear template |
| RG28 | CTAAGACATTTCATGGATGG | **Reverse** | |
| **For defining the clonal purity** | | | |
| RRG1591 | TATGGGCCTAAGATAGTATTACC | **Forward** | P1 |
| RRG97 | TCCGCAGGATGTTTCACATA | **Reverse** | P2 |
| RRG1602 | AAGACACACAAGAACATGACACTGCC | **Reverse** | P5 |
| **Detection of antisense RNA expression** | | | |
| RG3335 | TTTTGCTTCAGTCCAAACACT | **Forward** | P3 |
| RG3340 | GCACACAGTTATATATAATCGATAC | **Reverse** | P4 |
| **Assessment of flanking gene expression** | | | |
| RG26 | GCTTTGGATTGTTTGTCTTA | **Forward** | ECH_0230 RT-PCR |
| RG3337 | CCCTATTGCTCCTATAAGCA | **Reverse** | |
| RG3338 | TAGGATATTATCGTCCTTGTCT | **Forward** | ECH_0232 RT-PCR |
| RG3298 | AACTTCAGGGTTACAATCAAAC | **Reverse** | |
| **For RT-qPCR** | | | |
| RG2018 | AAATTGGGACGGAAGCG | **Forward** | p28-Omp19 (ECH_1143) |
| RG2019 | GCAAAACCTAAAAACGGG | **Reverse** | |
| RG2039 | TET/TCCTCCCCAAACGATGTATTCA/BHQ | **Probe** | |
| RG2020 | GACACTAATAGCGGTGAC | **Forward** | p28-Omp14 (ECH_1136) |
| RG2021 | ATGCCTTCATTTTTAAGGAC | **Reverse** | |
| RRG215 | FAM-TACTTTGGACTATCTCGTGAAGAC/BHQ | **Probe** | |
| RG3036 | GAATGTCCCTCCACAAGATG | **Forward** | RNase III |
| RG3037 | AAATGTAGGGTTGTGATCTGG | **Reverse** | |
| RG3038 | ACCAGCTCCTGCATATCACACCAT | **Probe** | |
| RG2180 | CAA GTC GAA CGG ACA AT | **Forward** | 16S |
| RG2181 | T TCT AAT GGC TAT TCC ATA CTA C | **Reverse** | |
| RG2182 | /56-FAM/CC CGT CTG C/ZEN/C ACT AAC AAT TAT TTA TAA CC/3IABkFQ/ | **Probe** | |
| **Detection of ECH_1143 RNA expression** | | | |
| RG3303 | TCATAACAAGTGCATTGATATCA | **Forward** | |
| RG2019 | GCAAAACCTAAAAACGGG | **Reverse** | |
| RG3304 | AAGCTCTATTCTTGGACCATC | **Reverse** | For cDNA synthesis |

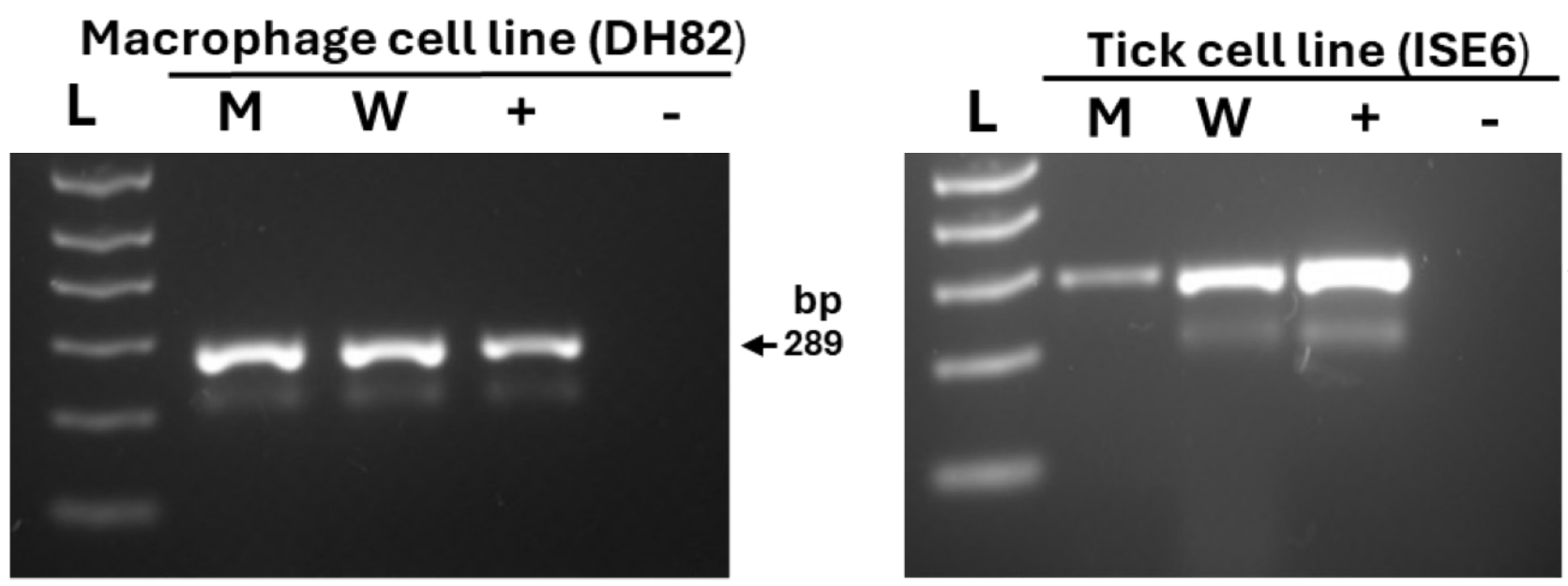


**Figure S1.** Analysis of *ECH_1143* sense RNA expression by RT-PCR. Expression of the *ECH_1143* sense transcript was assessed by RT-PCR using RNA isolated at 72 h post-infection from wild-type (W) and antisense mutant (M) *E. chaffeensis* grown in macrophage and tick cells. RNA inputs were normalized based on 16S rRNA levels determined by qRT-PCR prior to cDNA synthesis. First-strand cDNA was generated using primer RG3304 and subsequently amplified with primer pair RG3303 and RG2019. Genomic DNA controls (+) and a no-template control (−) were included to verify assay specificity.  DNA markers (1 kb Plus DNA ladder) (L) was used to define the amplicon sizes. Transcript abundance was evaluated by semi-quantitative PCR using 30 amplification cycles for samples from macrophage-derived cDNA and 35 cycles for the tick cell culture-derived cDNA. Comparable ECH_1143 transcript levels were observed for the mutant similar to wild-type in macrophage culture, whereas reduced transcript abundance was detected for the antisense mutant grown in the tick cell line. These data are in agreement with the qRT-PCR data presented in Fig 7 for the same time point assessed.
